# Comparative structures and evolution of vertebrate lipase H (LIPH) genes and proteins: a relative of the phospholipase A1 gene families

**DOI:** 10.1007/s13205-012-0087-z

**Published:** 2012-09-25

**Authors:** Roger S. Holmes, Laura A. Cox

**Affiliations:** 1 Department of Genetics, Texas Biomedical Research Institute, San Antonio, TX USA; 2 Southwest National Primate Research Center, Texas Biomedical Research Institute, San Antonio, TX, USA; 3 School of Biomolecular and Physical Sciences, Griffith University, Nathan, QLD 4111 Australia

**Keywords:** Vertebrates, Amino acid sequence, Lipase H, Evolution, Phylogeny

## Abstract

Lipase H (LIPH) is a membrane-bound phospholipase generating 2-acyl lysophosphatidic acid (LPA) in the body. LPA is a lipid mediator required for maintaining homeostasis of diverse biological functions and in activating cell surface receptors such as P2Y5, which plays an essential role in hair growth. Bioinformatic methods were used to predict the amino acid sequences, secondary and tertiary structures, and gene locations for *LIPH* genes and encoded proteins using data from several vertebrate genome projects. Vertebrate *LIPH* genes contained ten coding exons transcribed on either the positive or negative DNA strands. Evidence is presented for duplicated *LIPH* genes for the chicken and zebra fish genomes. Vertebrate LIPH protein subunits shared 56–97 % sequence identities and exhibited sequence alignments and identities for key LIPH amino acid residues as well as extensive conservation of predicted secondary and tertiary structures with those previously reported for horse pancreatic lipase (LIPP), with ‘N-signal peptide’, ‘lipase,’ and ‘plat’ structural domains. Comparative studies of vertebrate LIPH sequences with other phospholipase A1-like lipases (LIPI and PS-PLA1), as well as vascular and pancreatic lipases, confirmed predictions for LIPH N-terminal signal peptides (residues 1–18); a conserved vertebrate LIPH N-glycosylation site (66NVT for human LIPH); active site ‘triad’ residues (Ser 154; Asp 178; His 248); disulfide bond residues (233–246; 270–281; 284–292; 427–446), and a ‘short’ 12 residue ‘active site lid’, which is comparable to other phospholipases examined. Phylogenetic analyses demonstrated the relationships and potential evolutionary origins of the vertebrate *LIPH* family of genes related to, but distinct from other phospholipase A1-like genes (*LIPI* and *PS*-*PLA1*), and from vascular lipase and pancreatic lipase gene families.

**Electronic supplementary material:**

The online version of this article (doi:10.1007/s13205-012-0087-z) contains supplementary material, which is available to authorized users.

## Introduction

Lipase H (LIPH; E.C.2.7.11.30; also known as phosphatidic acid-selective phospholipase A1α or PA-PLA1alpha) is a membrane-bound phospholipase catalyzing the production of fatty acids and lysophosphatidic acid (LPA) (Jin et al. 2002; Sonoda et al. 2002; Aoki et al. 2007, 2008). LPA is a lipid mediator involved in diverse biological functions in the body (Moolenaar 1995). Most of these functions are mediated by G protein-coupled receptors (GPCRs) specific to LPA, for which six GPCRs have been described (Hama and Aoki 2010). Of these, PLA(6) (or P2Y5) (Yanagida et al. 2009) and LIPH have been identified as playing a major role in hair follicle cells, with autosomal recessive hypotrichosis (ARH) resulting from *LIPH* mutations, leading to the woolly hair hypotrichosis phenotype in human populations and sparse hair on the scalp (Aoki et al. 2008; Shimomura et al. 2009; Shinkuma et al. 2010). Two other related phospholipase A1-like enzymes have been reported in vertebrates, including lipase I (LIPI; E.C.2.7.11.34), also known as phosphatidic acid-selective phospholipase A1β, PA-PLA1β, cancer-testis antigen 17 (CT17) or LPD lipase (PLDL), which is a membrane-associated phospholipase catalyzing the production of fatty acids and lysophosphatidic acid (LPA) from phosphatidic acid (Hiramatsu et al. 2003; Wen et al. 2003), and a phosphatidylserine-specific phospholipase A1 (PS-PLA1), which hydrolyzes fatty acids at the sn-1 position of phosphatidylserine and 1-acyl-2-lysophosphatidylserine (Sato et al. 1997; Wen et al. 2003).

Other lipases are known to function in triglyceride metabolism in the body including lipoprotein lipase (LPL) (Wion et al. 1987; Holmes et al. 2011a), hepatic lipase (HL) (Martin et al. 1988; Holmes et al. 2011b), and endothelial lipase (EL) (Jaye et al. 1999; Cilingiroglu and Ballantyne 2004; Holmes et al. 2011c). These three enzymes are commonly referred to as the vascular lipase gene family due to their roles in triglyceride metabolism of circulating lipoproteins and in facilitating lipoprotein uptake into cells and tissues of the body (Brown and Rader 2007; Annema and Tietge 2011). Pancreatic lipase (PL) and related PL-like enzymes are a second major group of triglyceride lipases secreted from the exocrine pancreas playing major roles in lipid digestion (see Lowe 2002). Moreover, at least six mammalian acid lipase genes and proteins have also been described which represent a third group of triglyceride lipases including lysosomal lipase (LIPA), gastric lipase (LIPF), and epidermal cell lipases (LIPK, LIPM and LIPN) (see Holmes et al. 2010).

Human *LIPH* is expressed in several tissues of the body, including the intestine, lung, pancreas (Jin et al. 2002), prostate, testis, ovary, and platelets (Sonoda et al. 2002), and hair follicle cells, where a critical role for LPA signaling has been identified, facilitated by LIPH and the LPA receptor, P2Y5 (Inoue et al. 2011). Studies of genes controlling hair growth and scalp hair loss for several human populations (Ali et al. 2007; Jelani et al. 2008; Horev et al. 2009; Naz et al. 2009) have confirmed a link between *LIPH* mutations, genetically inherited hair loss, and congenital woolly hair/hypotrichosis (Shimomura et al. 2009; Shimomura 2012). Moreover, specific human founder gene *LIPH* mutations have been identified which involve key LIPH amino acid substitutions (Shinkuma et al. 2010), including a partial *LIPH* exon two duplication or an exon four deletion/insertion (Pasternack et al. 2009) and missense mutations causing autosomal recessive hypotrichosis (LAH2) (Naz et al. 2009). Knock-out *Liph* mice exhibited wavy hairs due to the aberrant formation of the hair follicle inner root sheath (Inoue et al. 2011) which confirmed the genetic link of *LIPH* with hair follicle development in this organism. In addition, an exon nine deletion of the *LIPH* gene has been shown to be responsible for the ‘rex’ hair coat phenotype in rabbits (Diribarne et al. 2011).

Structures for several human and animal *LIPH* genes and cDNA sequences have been determined, including human (*Homo sapiens*) (Jin et al. 2002; Sonoda et al. 2002; Hiramatsu et al. 2003), mouse (*Mus musculus*) (Wen et al. 2003; Carninci et al. 2005; Inoue et al. 2011), rat (*Rattus norvegicus*) (Gerhard et al. 2004), cow (*Bos taurus*) (Zimin et al. 2009), and chicken (*Gallus gallus*) (Hesse et al. 2012) *LIPH* genes. Human *LIPH*, which spans 44.4 kilobases (kbps) and comprises ten coding exons on the reverse strand, is localized on chromosome 3, near *MAP3K13*, encoding a member of the serine/threonine protein kinase family, and *SENP2*, encoding sentrin-specific peptidase 2 (Kent et al. 2003). Mouse *Liph* encodes also ten exons, spans 41.7 kbps on chromosome 16, and is expressed in high levels in placenta and colon (Su et al. 2004; Carninci et al. 2005).

There have been few biochemical and structural studies of mammalian LIPH; however, Jin et al. (2002) have reported amino acid sequences for human and mouse LIPH, derived from cDNA sequences, encoding 451 amino acids containing N-terminal and lipase domains in each case. Human LIPH exhibited 85 % identity with mouse LIPH and 46–47 % identity with vascular lipase sequences (LPL, EL and HL) and phosphatidic acid-preferential PLA1β (LIPI or PS-PLA1beta). Studies of amino acid sequences arising from *LIPH* founder mutations from individuals with autosomal recessive hypotrichosis have enabled the identification of key residues for this enzyme, including the active site and cysteine residues, forming intramolecular disulphide bonds (Shinkuma et al. 2010).

This paper reports the predicted gene structures and amino acid sequences for several vertebrate *LIPH* genes and proteins, the predicted secondary and tertiary structures for vertebrate LIPH protein subunits, and the structural, phylogenetic and evolutionary relationships for these genes and enzymes with related vertebrate phospholipase A1-like enzymes (LIPI and PS-PLA1), and with vascular and pancreatic lipase gene families.

## Methods

### Vertebrate *LIPH* gene and protein identification

Basic local alignment search tool (BLAST) studies were undertaken using web tools from the National Center for Biotechnology Information (NCBI) (http://www.blast.ncbi.nlm.nih.gov/Blast.cgi) (Altschul et al. 1997). Protein BLAST analyses used vertebrate LIPH amino acid sequences previously described (Table 1). Non-redundant protein sequence databases for several vertebrate genomes were accessed from sources previously described (Holmes et al. 2011a). Predicted LIPH-like protein sequences were obtained in each case and subjected to analyses of predicted protein and gene structures.

**Table 1:** Vertebrate *LIPH*, *LIPI* and *PS*-*PLA1* genes and protein subunits

Vertebrate	Species	Gene	RefSeq ID prediction^1,2^	GeneBank ID	Chromosome location	Coding exons (strand)	Gene size (bps)	UNIPROT ID	Amino acids	Subunit MW	Pl
Human	*Homo Sapiens*	*LIPH*	NM_139248.2	BC064941	3:186,709,275–186,752,953	10 (+ve)	44,800	Q8WW8	451	50,859	7.2
Chimp	*Pan troglodytes*	*LIPH*	XP_516924.2¹	NA	3:191,061,117–191,090,382	10 (−ve)	29,266	Na	451	50,859	7.2
Orangutan	*Pongo abelii*	*LIPH*	ENSPPYT16713²	NA	3:189,387,154–189,421,133	10 (−ve)	33,980	Na	451	50,897	7.1
Rhesus	*Macaca mulatta*	*LIPH*	XP_001090044.1¹	NA	2:101,085,592–101,143,467	10 (+ve)	57,876	Na	450	51,582	8.8
Mouse	*Mus musculus*	*Liph*	NM_001083894.1	BC037489	16:21,956,166–21,984,342	10 (−ve)	28,177	Q8CIV3	451	50,675	6.8
Rat	*Rattus norvegicus*	*Liph*	NM_001044279	BC062045	11:77,895,628–77,940,437	Na (+ve)	44,809	Q32PY2	451	50,826	8.3
Guinea Pig	Cavia porcellus	*LIPH*	XP_003476975.1¹	NA	7:50,635,497–50,661,027	10 (−ve)	25,531	Na	461	51,718	7.5
Horse	*Equus caballus*	*LIPH*	XP_001497826.1¹	NA	19:23,706,899–23,736,710	10 (−ve)	29,812	Na	451	50,649	7.9
Dog	*Canis familiaris*	*LIPH*	XP_545236.2¹	NA	34:21,278,626–21,305,983	10 (−ve)	27,358	Na	456	51,393	8.9
Panda	*Ailuropoda melanoleuca*	*LIPH*	XP_002915705.1¹	NA	GL192440:723,725–749,447	10 (+ve)	25,723	Na	451	50,948	8.8
Cow	*Bos taurus*	*LIPH*	XP_589466.3¹	NA	1:83,522,271–83,560,671	10 (+ve)	38,401	Na	476	50,348	7.1
Rabbit	*Oryctolagus cuniculus*	*LIPH*	NM_001082106.1	AF351188	14:80,045,888–80,094,405	10 (+ve)	48,518	Q9BDJ4	452	51,136	8.4
Opossum	*Monodelphis domestica*	*LIPH*	ENSMODP3338¹	NA	2:541,400,320–541,423,098	10 (−ve)	39,845	Na	446	50,518	8.6
Chicken	*Gallus gallus*	*LIPH1*	XP_416675.2¹	NA	1:101,409,883–101,425,858	10 (−ve)	15,976	Na	463	52,907	8.4
Chicken	*Gallus gallus*	*LIPH2*	XP_422687.3¹	NA	9:4,675,149–4,684,014	10 (−ve)	8,866	Na	459	51,109	6.5
Lizard	*Anolis carolensis*	*LIPH*	XP_003224081.1¹	NA	GL343230:1,571,716–1,604,227	11 (−ve)	32,512	Na	442	49,455	7.9
Frog	*Xenopus tropicalis*	*LIPH*	NP_001011098.1	BC084493	9:15,825–32,184	10 (−ve)	16,360	Q5XGE9	460	52,170	6.6
Zebrafish	*Danio rerio*	*LIPH1*	NM_001003499.1	BC078354	21:33,911,120–33,933,839	10 (−ve)	22,720	Q6DBU8	454	51,811	8.4
Zebrafish	*Danio rerio*	*LIPH2*	XP_001342691.1¹	NA	24:22,665,923–22,680,183	10 (+ve)	14,261	Na	448	50,663	7.2
Human	*Homo Sapiens*	*LIPI*	NM_198996.2	BC140336	21:14,403,188–14,501,115	10 (−ve)	98,120	Q6XZB0	481	55,318	9.2
Mouse	*Mus musculus*	*Lipi*	NM_177142	BC147403	16:75,541,302–75,586,195	11 (−ve)	44,894	Q8BVB7	476	54,234	8.9
Rat	*Rattus norvegicus*	*Lipi*	NM_001105899.1	NA	11:14,336,907–14,375,937	11 (−ve)	39,031	Na	476	54,257	9.0
Horse	*Equus caballus*	*LIPI*	XP_001498634.2¹	NA	26:13,392,409–13,454,760	10 (−ve)	62,352	Na	459	51,703	8.7
Human	*Homo Sapiens*	*PS*-*PLA1*	NM_001206960.1	BC044703	3:119,316,761–119,348,312	11 (+ve)	31,552	Q53H76	456	49,715.0	7.1
Mouse	*Mus musculus*	*Ps*-*Pla1*	NM_134102.4	BC003470	16:38,396,540–38,432,956	11 (−ve)	36,417	Q8VI78	456	49,983.0	8.3
Rat	*Rattus norvegicus*	*Ps*-*Pla1*	NM_138882.1	BC078727	11:64,099,850–64,137,017	11 (+ve)	37,168	P97535	456	50,202	8.3
Chicken	*Gallus gallus*	*PS*-*PLA1*	XP_001233532.1¹	NA	1:79,608,797–79,625,612	11 (−ve)	16,816	Na	456	50,069.0	8.8
Zebrafish	*Danio rerio*	*PS*-*PLA1*	NM_207056.1	BC155819	9:22,248,524–22,257,714	11 (+ve)	9,191	A9JRW4	462	51,225.0	6.1

*RefSeq* NCBI reference sequence, ¹ predicted NCBI sequence, ² predicted UCSC Genome Browser sequence, *NA* not available, *GL* gene scaffold ID, *pI* isoelectric point, *bps* base pairs of nucleotide sequence

BLAST-like alignment tool (BLAT) analyses were subsequently undertaken for each of the predicted vertebrate LIPH amino acid sequences using the University of California Santa Cruz (UCSC) genome browser (http://www.genome.ucsc.edu/cgi-bin/hgBlat) (Kent et al. 2003) with the default settings to obtain the predicted locations for each of the vertebrate *LIPH* genes, including predicted exon boundary locations and gene sizes. This browser was also used to show alignments of *LIPH* genes from several vertebrate genomes (called Multiz alignments). Structures for human, mouse, and rat *LIPH* isoforms were obtained using the AceView website (http://www.ncbi.nlm.nih.gov/IEB/Research/Acembly/index.html?human) to examine predicted gene and protein structures (Thierry-Mieg and Thierry-Mieg 2006). Vertebrate LIPH sequences were aligned using the ClustalW2 multiple sequence alignment program (http://www.ebi.ac.uk/Tools/clustalw2/index.html).

### Predicted structures and properties of vertebrate LIPH protein subunits

Predicted secondary and tertiary structures for vertebrate LIPH-like subunits were obtained using the PSIPRED v2.5 web site tools (http://www.bioinf.cs.ucl.ac.uk/psipred/psiform.html) (McGuffin et al. 2000) and the SWISS model web tools (swissmodel.expasy.org), respectively (Kopp and Schwede 2004). The reported tertiary structure for horse pancreatic lipase (PTL) (PDB: 1hpl) (Bourne et al. 1994) served as the reference for the predicted rabbit, chicken, and zebrafish LIPH tertiary structures, with a modeling range of residues 38 to 447. Theoretical isoelectric points and molecular weights for vertebrate LIPH subunits were obtained using Expasy web tools (http://www.au.expasy.org/tools/pi_tool.html). SignalP 3.0 web tools were used to predict the presence and location of signal peptide cleavage sites (http://www.cbs.dtu.dk/services/SignalP/) for each of the predicted vertebrate LIPH sequences. The NetNGlyc 1.0 Server was used to predict potential N-glycosylation sites for vertebrate LIPH subunits (http://www.cbs.dtu.dk/services/NetNGlyc/). The Mobyle Server (http://www.mobyle.pasteur.fr) was used to predict phospholipid binding sites for vertebrate LIPH sequences.

### Comparative human (*LIPH*) and mouse (*Liph*) tissue expression

The UCSC genome browser (http://www.genome.ucsc.edu) (Kent et al. 2003) was used to examine GNF1 expression atlas 2 data using various expression chips for human *LIPH* and mouse *Liph* genes, respectively (http://www.biogps.gnf.org) (Su et al. 2004). Gene array expression ‘heat maps’ were examined for comparative gene expression levels among human and mouse tissues showing high (red), intermediate (black), and low (green) expression levels.

### Phylogenetic studies and sequence divergence

Alignments of vertebrate LIPH, LIPI, PS-PLA1, human, mouse, and zebrafish HL (hepatic lipase), LPL (lipoprotein lipase), EL (endothelial lipase) protein sequences, as well as human, mouse, and frog pancreatic lipases (PL) sequences were assembled using BioEdit v.5.0.1, as previously described (Holmes et al. 2011a). Alignment ambiguous regions were excluded prior to phylogenetic analysis yielding alignments of 349 residues for comparisons of sequences (Table 1). Evolutionary distances were calculated using the Kimura option as previously described (Holmes et al. 2011a). Phylogenetic trees were constructed from evolutionary distances using the neighbor-joining method (Saitou and Nei 1987). Tree topology was reexamined by the boot-strap method (100 bootstraps were applied) of resampling and only values that were highly significant (≥95) are shown.

## Results and discussion

### Alignments of human and other vertebrate LIPH subunits

**Fig. 1:**
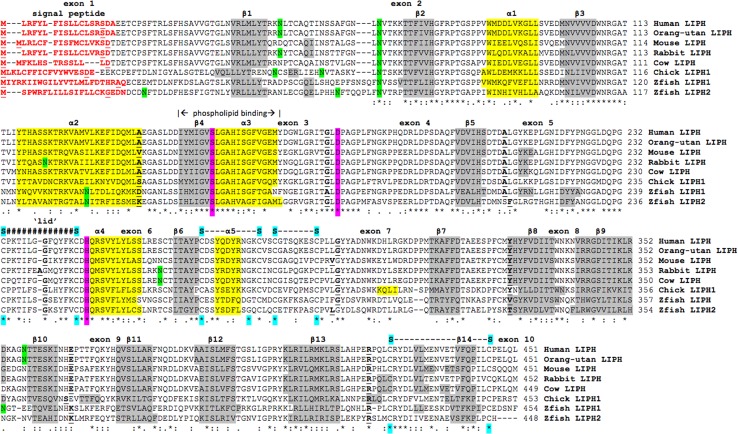
Amino acid sequence alignments for human and other vertebrate LIPH subunits. See Table 1 for sources of LIPH sequences; *asterisk* shows identical residues for LIPH subunits—similar alternate residues; dissimilar alternate residues; N-signal peptide residues are in *red*; N-glycosylation residues are in *green*; active site triad residues Ser; Asp; and His are in *pink*; disulfide bond Cys residues are in *blue*; predicted helix; predicted sheet; *bold* font shows known or predicted exon junctions; exon numbers refer to human LIPH gene; predicted ‘lid’ covering the active site (human LIPH residues 234–245) are shown *hashes*; *Hu* human LIPH; *Or* orangutan LIPH; *Mo* mouse LIPH; *Rb* rabbit LIPH; *Co* cow LIPH; *Ch* chicken LIPH; *Z1* zebrafish LIPH1; and *Z2* zebrafish LIPH2

**Fig. 2:**
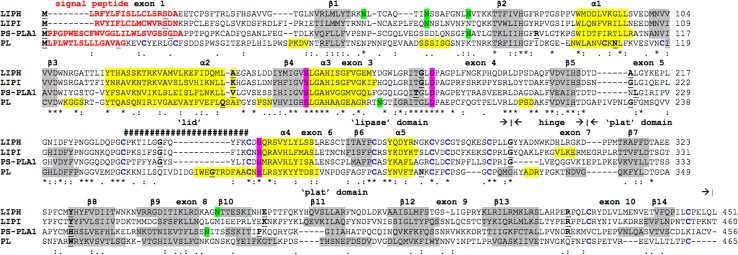
Amino acid sequence alignments for human LIPH, lipase I (LIPI), phosphatidylserine-specific phospholipase A1 (PS-PLA1) and pancreatic lipase (PL). See Table 1 and Table 1s for sources of human LIPH, lipase I (LIPI), phosphatidylserine-specific phospholipase A1 (PS-PLA1) and pancreatic lipase (PL) sequences; *asterisk* shows identical residues for subunits—similar alternate residues; dissimilar alternate residues; N-signal peptide residues are in *red*; known or predicted N-glycosylation residues are in *green*; active site triad residues Ser; Asp; and His are in *pink*; disulfide bond Cys residues are shown (**C**); predicted helix; predicted sheet; *bold* font shows known or predicted exon junctions; exon numbers refer to human LIPH gene; residues proposed for location in the hinge region are shown; predicted ‘lid’ residues covering the active site (human LIPH residues 234–245) are shown *hashes*; major ‘lipase’, ‘lid’ and ‘plat’ domains are shown

The deduced amino acid sequences for orangutan (*Pongo abelii*), rabbit (*Oryctolagus cuniculus*), cow (*Bos taurus*), and chicken (*Gallus gallus*) LIPH subunits and for zebrafish (*Danio rerio*) LIPH1 and LIPH2 subunits are shown in Fig. 1 together with the previously reported sequences for human (*Homo sapiens*) and mouse (*Mus musculus*) LIPH subunits (Table 1) (Jin et al. 2002; Sonoda et al. 2002). Alignments of the human and other vertebrate LIPH subunits examined in this figure showed between 47 and 99 % sequence identities (Table 2), suggesting that these are products of the same family of genes and proteins (Table 2). The amino acid sequence for human LIPH contained 451 residues while other vertebrate LIPH subunits contained 442 (lizard LIPH: *Anolis carolensis*) to 460 residues (frog LIPH: *Xenopus tropicalis*) (Fig. 1; Table 1). Several key amino acid residues for vertebrate LIPH were recognized (sequence numbers refer to human LIPH) (Fig. 1). These include the hydrophobic N-terminus signal peptide (residues 1–18), as reported for rat hepatic lipase (Laposata et al. 1987); the catalytic triad for the active site (Ser 154; Asp 178; His 248) (Fig. 1s) forming a charge relay network for substrate hydrolysis, similar to human lipoprotein lipase (Emmerich et al. 1992); a predicted phospholipid binding site containing the active site Ser 154 (Fig. 2s); and disulfide bond forming residues (Cys233/Cys246; Cys270/Cys281; Cys284/Cys292; and Cys427/Cys446) (Jin et al. 2002). Identical residues were observed for each of the vertebrate LIPH subunits for the active site triad and disulfide bond forming residues; however, the N-terminus 18-residue signal peptide underwent changes in sequence but retained predicted signal peptide properties (Fig. 1; Table 1). One of the N-glycosylation sites (designated as site 5) predicted for human LIPH at Asn 66–Val 67–Thr 68 was retained for each of the 18 vertebrate LIPH sequences examined; however, predicted N-glycosylation sites were observed at other positions for some sequences, including for example, Asn 50–Leu 51–Thr 52 (site 2) and Asn 357–Thr 358–Thr 359 (site 14), for human LIPH (Table 2). Given the reported role of the N-glycosylated carbohydrate group in contributing to the stability and maintaining catalytic efficiency of a related enzyme (human carboxylesterase or CES1) (Kroetz et al. 1993), this property may be shared by vertebrate LIPH as well, especially for those containing multiple predicted sites for N-glycosylation, such as dog LIPH, which contains four such sites.

**Table 2:** Known or predicted N-glycosylation sites for vertebrate LIPH subunits

Vertebrate	Site 1	Site 2	Site 3	Site 4	Site 5	Site 6	Site 7	Site 8	Site 9	Site 10	Site 11	Site 12	Site 13	Site 14	Site 14	Site 15	No of sites
LIPH																	
Human		**50NLT**	58NSS		**66NVT**										**357NTT**		**3**
Chimp		**50NLT**	58NSS		**66NVT**										**357NTT**		**3**
Orangutan		**50NLT**	58NSS		**66NVT**										**357NTT**		**3**
Rhesus		**53NLT**	61NSS		**69NVT**										**361NTT**		**3**
Mouse			58NST		**66NVT**						262NCS				357NIT		**1**
Rat			58NST		**66NVT**						262NCS				357NIT		**1**
Cow			54NST		**62NVT**						**258NCT**				353NTT		**2**
Horse			58NPT		**66NVT**		**122NKT**								357NTT		**2**
Dog		55NQT	**63NST**		**71NMT**						**267NCS**				**362NIT**		**4**
Rabbit		**50NYT**	58NST		**66NVT**		**122NKT**				**263NCT**			340NKS	358NTT		**4**
Opossum					**66NVT**										365NYT		**1**
Chicken 1		**62NCS**	**71NVT**		**79NTS**						277NIT				351NKS		**3**
Chicken 2			**59NST**		**75NVT**		120NAS				265NIT					389NIT	**1**
Lizard		**68NLT**	**76NVT**	79NLT	**87NVT**						**255NVT**				348NTT	379NIS	**4**
Frog			67NST		**75NVT**					**177NGS**		289NCT			366NVT		**2**
Zebrafish 1					**73NVS**			**137NLT**	151NLS		267NGC				**358NGT**		**3**
Zebrafish 2	**25NFT**		**62NFT**		**70NVT**			**134NIT**			264NRT				358NVT		**4**
Tetraodon					**71NFS**	**95NIT**		**135NLT**		173NGS			**315NQT**				**4**

The identified N-glycosylation site is for human LIPH (Jin et al. 2002)

Amino acid residues are shown for known or predicted N-glycosylation sites: *N* Asn; *A* Ala; *T* Thr; *S* Ser; *M* Met; *L* Leu; *D* Asp; *G* Gly; *F* Phe; *I* Ile; *V* Val

Sites with high probabilities for N-glycosylation are highlighted in bold

### Alignments of human LIPH with other lipase subunits

Alignments of human LIPH (Jin et al. 2002), lipase I (LIPI) (Hiramatsu et al. 2003; Wen et al. 2003), phosphatidylserine-specific phospholipase A1 (PS-PLA1) (Sato et al. 1997; Wen et al. 2003) and pancreatic lipase (PL) (Winkler et al. 1990; Lowe 2002) sequences are shown in Fig. 2. The following key amino acid residues were observed for each of these lipases consistent with those observed for the mammalian LIPH sequences (see Table 1). These included the N-terminal signal peptide sequences, which were distinct for each lipase but retained the predicted signal peptide functional role; the active site triad residues aligning with LIPH Ser 154, Asp 178, and His 248; the human LIPH disulfide cysteine residues (Cys 233/Cys 246; Cys 270/Cys 281; Cys 284/Cys 292; and Cys 427/Cys 446); however, human PS-PLA1 did not contain the last disulfide pair, while additional disulfide bonds were observed for human pancreatic lipase (corresponding to human PL Cys 20/Cys 26; Cys 106/Cys 118); distinct N-glycosylation sites were predominantly observed for each of the lipase sequences examined, although the human LIPH Asn 66–Val 67–Thr 68 N-glycosylation site was also shared with the human PS-PLA1 sequence; the active site ‘lid’ sequences showed that LIPH contained fewer residues (12 amino acids) (Fig. 1s), in comparison with other lipases [EL (19 residues); LPL and HL (22 residues); and PL (23 residues); and a high basic amino acid content region was observed for human LIPH residues (Lys 303 → Lys 315)] which aligned proximate to a heparin-binding site (human EL 324 Lys → 333 Lys) reported for human EL, which binds this lipase to the heparan sulfate proteoglycans on the luminal side of endothelial cells (Hill et al. 1998). This high basic amino acid content region for the human LIPH sequence may contribute to heparin binding reported for this enzyme (Hiramatsu et al. 2003).

### Predicted secondary and tertiary structures of vertebrate LIPH subunits

Predicted secondary structures for vertebrate LIPH sequences were compared in Fig. 1, and similar α-helix and β-sheet structures were observed for all of the vertebrate LIPH subunits examined. Consistent structures were particularly apparent near key residues or functional domains including the β-sheet and α-helix structures near the active site Ser 154 (β4/α3); the active site His 248 (α4) residue; and the conserved N-glycosylation site at Asn 66–Val 67–Thr 68 (near β2). The primary structures for vertebrate LIPH sequences were also screened for candidate amphiphilic α-helices for forming of protein-phospholipid contacts using a bioinformatics method for predicting phospholipid-binding sites for smooth muscle caldesmon and calponin (Bogatcheva and Gusev 1995). Residues 147–167 for mouse LIPH (containing the active site Ser 154) were identified with an α-helical structure showing a high level of hydrophobicity which is a likely binding region at the active site for binding phospholipid substrates and in binding LIPH to membranes (Fig. 2s). Twelve amino acids were also identified near the active site histidine (His 248) and between two residues (Cys 233/Cys 246) forming disulfide intramolecular bonds as the proposed ‘active site lid’ structure for vertebrate LIPH. In addition, eight β-sheets were observed at the LIPH C-terminus end, which is consistent with PLAT domain structures previously reported for horse pancreatic lipase (PTL) (Bourne et al. 1994). It is also apparent from these studies that the LIPH subunits examined have highly similar secondary structures to those of the other A1-like phopholipases (LIPI and PS-PLA1) examined.

**Fig. 3:**
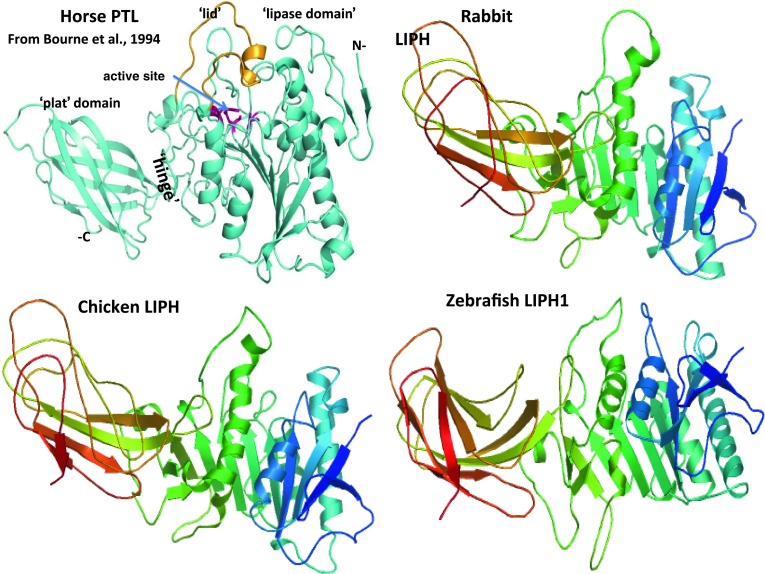
Tertiary structure for horse pancreatic lipase (PL) and predicted tertiary structures for rabbit, chicken and zebrafish LIPH subunits The tertiary structure for horse pancreatic lipase (PL) is from Bourne et al. 1994; predicted rabbit, chicken and zebrafish LIPH 3-D structures were obtained using the SWISS MODEL web site http://www.swissmodel.expasy.org and based on the reported structure for horse PL (PDB: 1hpl); the *rainbow color code* describes the 3D structures from the N- (*blue*) to C-termini (*red color*); N refers to amino-terminus; C refers to carboxyl terminus; the ‘lipase’ and ‘plat’ domains, the active site region and the ‘lid’ covering the active site are shown

Figure 3 describes predicted tertiary structures for rabbit, chicken, and zebrafish LIPH sequences, in comparison with horse pancreatic lipase (PL) (Bourne et al. 1994). Identification of specific structures within the predicted LIPH sequences were based on the reported structure for horse PL which identifies a sequence of twisted β-sheets interspersed with several α-helical structures which are typical of the alpha–beta hydrolase super-family. The active site LIPH triad was centrally located which is similar to that observed for other lipases (Bourne et al. 1994) and carboxylesterase (human CES1) (Bencharit et al. 2003). The major difference between LIPH and other lipases examined (Fig. 3) is the much smaller size of the ‘lid’ region at positions 234–246, which may act as a surface loop that partially covers the opening to the catalytic triad and allows access to the active site by LIPH substrates. This ‘lid’ structure is readily apparent in the predicted structures for rabbit, chicken, and zebrafish LIPH. These comparative studies of vertebrate LIPH proteins suggest that the properties, structures, and key sequences are substantially retained for the vertebrate sequences examined.

### Predicted gene locations and exonic structures for vertebrate *LIPH* genes

Table 1 summarizes the predicted locations for vertebrate *LIPH* genes based upon BLAT interrogations of several vertebrate genomes using the reported sequences for human and mouse LIPH (Jin et al. 2002; Sonoda et al. 2002) and the predicted sequences for other vertebrate LIPH proteins and the UCSC genome browser (Kent et al. 2003). Human and mouse *LIPH* genes were located on human chromosome 3 and mouse chromosome 16, with distinct gene locations to those for other phospholipases, including lipase I (*LIPI*) (human chromosome 21 and also on mouse chromosome 16) and phosphatidylserine-specific phospholipase A1 (PS-PLA1) (also on human chromosome 3 and mouse chromosome 16) (Table 1; Table 1s). The chicken (*Gallus gallus*) and zebrafish (*Danio rerio*) genome showed evidence of duplicated *LIPH* genes, with predicted *LIPH1* and *LIPH2* genes being located on separate chromosomes, in each case (Table 1). This is consistent with many other gene duplication events during zebrafish evolution that have occurred predominantly by polyploidisation or duplication of large chromosomal segments (Postlethwait et al. 1998).

**Fig. 4:**
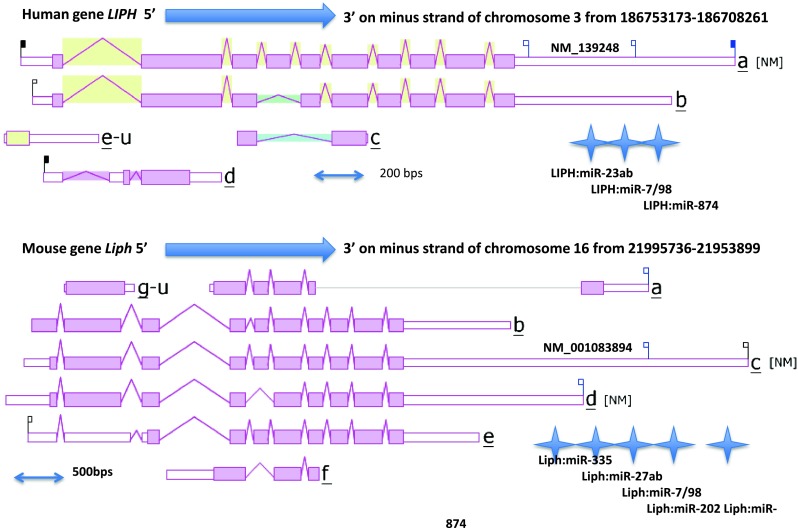
Gene structures and isoforms for the human and mouse *LIPH* genes. Derived from AceView website http://www.ncbi.nlm.nih.gov/IEB/Research/Acembly/ (Thierry-Mieg and Thierry-Mieg 2006); the major isoform variants are shown with capped 5′- and 3′-ends for the predicted mRNA sequences; introns and coding exons are shown; miRNA binding sites were identified for the *LIPH* genes (shown as *stars*); the direction for transcription is shown; *3′-UTR* 3′-untranslated region; a *scale* is shown in base pairs (bps); coding exons are in *pink*; untranslated 5′- and 3′-regions are shown as *open rectangles*

Figure 1 summarizes the predicted exonic start sites for several vertebrate *LIPH* genes with each having ten exons, in identical or similar positions to those reported for the human *LIPH* and mouse *Liph* genes (Thierry-Mieg and Thierry-Mieg 2006). Human *LIPI*, *PLA1A,* and *PL* (encoding pancreatic lipase) genes contained 10, 11, and 12 exons, respectively, which are in similar positions for several exons of vertebrate *LIPH* genes, suggesting that these are related genes (Fig. 2). Figure 4 illustrates the structures for human *LIPH* and mouse *Liph* transcripts, with the major transcript isoforms being designated as NM_139248 and NM_001083894, respectively (Kent et al. 2003). The transcripts were 2.5 and 3.8 kb in length, respectively, with nine introns and ten exons in each case. The human *LIPH* genome sequence contained three microRNA sites (miR23ab; miR-7/98; and miR-874) located in the 3′-untranslated region, whereas the mouse *Liph* gene sequence contained five such sites (miR-335; miR-27ab; miR-7/98; miR-202; and miR-874). These microRNA-binding sites are potentially of major significance for the regulation of this gene. MicroRNAs are small noncoding RNAs that regulate mRNA and protein expression and have been implicated in regulating gene expression during embryonic development (Stefani and Slack 2008). Important associations for miRNAs have been recently reported for psoriasis, a chronic inflammatory human skin disease (Zibert et al. 2010). One such association included an overexpression of miR-221/2, leading to decreased TIMP3 (metalloproteinase inhibitor) (Uría et al. 1994) levels in the skin. Zibert et al. (2010) have also identified 42 upregulated miRNAs and five downregulated miRNAs in psoriatic skin, although *LIPH* mRNA targets were not among those differentiated between healthy and diseased skin.

Figure 3s shows a UCSC genome browser comparative genomics track that shows evolutionary conservation and alignments of the nucleotide sequences for the human *LIPH* gene, including the 5′-flanking, 5′-untranslated, intronic, exonic, and 3′-untranslated regions of this gene, with the corresponding sequences for eight vertebrate genomes, including four eutherian mammals (e.g. mouse, dog), a marsupial (opossum), a bird (chicken), frog, and zebrafish genomes. Extensive conservation was observed among these genomic sequences, particularly for the rhesus *LIPH* gene and for other eutherian mammalian genomes. In contrast with the eutherian mammalian genomes examined, other vertebrate genomes retained conserved sequences only within several of the exonic *LIPH* regions, particularly for exons two–six, which are conserved within zebrafish *LIPH* genes. It would appear that exon one, which encodes the ‘signal peptide’ domain *LIPH* nucleotide sequences, has undergone more extensive divergence than for the other exons, which have been predominantly conserved throughout vertebrate evolution.

### Comparative human and mouse *LIPH* tissue expression

Figure 4s presents ‘heat maps’ showing comparative *LIPH* gene expression for various human and mouse tissues obtained from GNF expression atlas data using the GNF1H (human) and GNF1M (mouse) chips (http://www.genome.ucsc.edu; http://www.biogps.gnf.org) (Su et al. 2004). These data supported a wide tissue expression for human *LIPH* and mouse *Liph* and a higher expression level in mouse pancreas, intestine, stomach, placenta, prostate, and thyroid tissues. Previous studies have reported significant human *LIPH* expression in various tissues, including the intestine, lung, pancreas (Jin et al. 2002), prostate, testis, ovary, and platelets (Sonoda et al. 2002), and hair follicle cells, where a critical role for LPA signaling has been identified, facilitated by LIPH and the LPA receptor, P2Y5 (Inoue et al. 2011). Diribarne et al. (2011) have examined *LIPH* expression in rabbit skin and hair follicles and reported higher expression levels in the outer hair root sheath than in the inner hair root sheath.

### Phylogeny and divergence of vertebrate *LIPH* and other lipase sequences

**Fig. 5:**
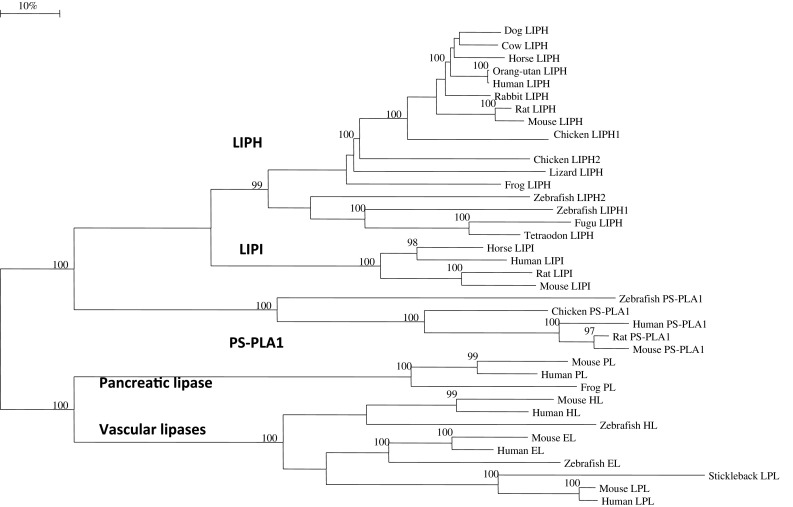
Phylogenetic tree of vertebrate LIPH sequences with mammalian LIPI and vertebrate PS-PLA1, hepatic lipase (HL), endothelial lipase (EL) and lipoprotein lipase (LPL), and pancreatic lipase (PL) amino acid sequences The tree is labeled with the gene name and the name of the vertebrate. Note the major cluster for the vertebrate LIPH sequences and the separation of these sequences from mammalian LIPI and from vertebrate PS-PLA1, HL, EL, LPL and PL sequences. See Table 1 and Table 1s for details of sequences and gene locations. A genetic distance scale is shown (% amino acid substitutions). The number of times a clade (sequences common to a node or branch) occurred in the bootstrap replicates are shown. Only replicate values of 95 or more, which are highly significant, are shown with 100 bootstrap replicates performed in each case. Note the four major lipase gene clusters: LIPH; LIPI; PS-PLA1; PL; and the vascular lipases (EL, HL and LPL). The phylogram is indicative of a closer evolutionary relationship between *LIPH* and *LIPI* phospholipase gene families

A phylogenetic tree (Fig. 5) was calculated by the progressive alignment of human and other vertebrate LIPH amino acid sequences with human, horse, rat, and mouse LIPI sequences: human, mouse, rat, chicken, and zebrafish PS-PLA1 sequences; human, mouse and zebrafish hepatic lipase (HL), and endothelial lipase (EL) sequences; human, mouse, and stickleback (a bony fish species) lipoprotein lipase (LPL) sequences; and human, mouse, and frog pancreatic lipase (PL) sequences. The phylogram showed clustering of the vertebrate LIPH sequences which were distinct from the mammalian LIPI sequences and the vertebrate PS-PLA1 sequences, as well as the vertebrate vascular lipase (HL, EL and LPL) and pancreatic lipase (PL) vertebrate lipase families. In addition, the zebrafish LIPH1 and LIPH2 sequences showed clustering within the fish LIPH sequences examined, which is consistent with these genes being products of a recent duplication event during fish evolution. Overall, these data suggest that the vertebrate *LIPH* gene arose from a gene duplication event of an ancestral phospholipase-like gene, resulting in at least three separate lines of phospholipase gene evolution, namely *LIPH*, *LIPI,* and the *PS*-*PLA1* genes. In addition, it is proposed that the mammalian LIPI gene may have arisen from a *LIPH* gene duplication event for which duplicated *LIPH1* and *LIPH2* genes were observed for the chicken genome. It is also suggested that these A1-like phospholipase genes (*LIPH*, *LIPI* and *PS*-*PLA1*) may have shared a common evolutionary ancestor with the vascular gene families (hepatic lipase, endothelial lipase and lipoprotein lipase) and the pancreatic lipase gene family. This is supported by the comparative biochemical and genomic evidence for vertebrate *LIPH*, *LIPI*, *PS*-*PLA1,* and the vascular and pancreatic lipase-like genes and encoded proteins, which share several key features of protein and gene structure, including having similar alpha–beta hydrolase secondary and tertiary structures.

## Conclusions

The results of this study suggest that vertebrate *LIPH* genes and encoded LIPH enzymes represent a distinct alpha–beta hydrolase-like gene and enzyme family which share key conserved sequences with other A1-like phospholipases (LIPI and PS-PLA1) and with structures that have been reported for the human lipase gene families, namely hepatic lipase, endothelial lipase, lipoprotein lipase and pancreatic lipase. LIPH is a major membrane-bound phosphatidic acid-selective phospholipase catalysing the production of fatty acids and lysophosphatidic acid. Bioinformatic methods were used to predict the amino acid sequences, secondary and tertiary structures and gene locations for *LIPH* genes and encoded proteins using data from several vertebrate genome projects. Evidence is presented for duplicated *LIPH* genes for the zebrafish and chicken genomes. Vertebrate LIPH protein subunits shared 56–97 % sequence identities and exhibited sequence alignments and identities for key LIPH amino acid residues as well as extensive conservation of predicted secondary and tertiary structures with those previously reported for mammalian pancreatic lipases, with ‘N-signal peptide’, ‘lipase’ and ‘plat’ structural domains. Phylogenetic analyses demonstrated the relationships and potential evolutionary origins of the vertebrate *LIPH* family of genes from a common ancestral gene with other A1-like phospholipase genes (*LIPI* and *PS*-*PLA1*), and with vascular lipase genes, hepatic lipase (*HL*), endothelial lipase (*EL*), and lipoprotein lipase (*LPL*), which were related to, but distinct from pancreatic lipase gene families. These studies also indicated that *LIPH* genes appeared early in vertebrate evolution prior to the teleost fish common ancestor and may have served as a source for the mammalian *LIPI* gene following an earlier gene duplication event.

### Dedication to Emeritus Professor Colin Masters

Emeritus Professor Colin Masters passed away recently following an extended illness with cancer on 6th April 2012. Colin had published extensively in internationally peer reviewed journals and other respected monograph publishing houses in biochemistry, molecular biology and biotechnology. Colin Masters has left a rich legacy and an outstanding research record for his former graduate students, postdoctoral researchers and collaborators to follow and admire. An obituary for Emeritus Professor Colin Masters is published in this three biotech volume outlining his research career and several of his achievements in academia and scientific research. One of us (RSH) benefited from his calm and expert research postgraduate and postdoctoral supervision and remained a friend, colleague and collaborator for nearly 50 years. This paper on vertebrate lipase H (LIPH) is dedicated to his memory, to an outstanding research career in Australian biochemistry and biotechnology and to the dedication and friendship he showed to so many of us who were supervised, helped and nurtured during his long and productive life.

## Electronic supplementary material

Below is the link to the electronic supplementary material.

Supplementary material 1 (PPTX 154 kb)

Supplementary material 2 (PPTX 110 kb)

Supplementary material 3 (PPTX 238 kb)

Supplementary material 4 (PNG 124 kb)

Supplementary material 5 (XLS 39 kb)

Supplementary material 6 (XLSX 11 kb)
