# Supplementary material for: Comparative structures and evolution of vertebrate lipase H (LIPH) genes and proteins: a relative of the phospholipase A1 gene families
Source: 3 Biotech. 2012 Sep 25;2(4):263–75. doi: 10.1007/s13205-012-0087-z (PMC3482443; doi:10.1007/s13205-012-0087-z)
Supplement: Supplementary file 1 — Supplementary material 1 (PPTX 154 kb) [file 13205_2012_87_MOESM1_ESM.pptx]

## Slide 1
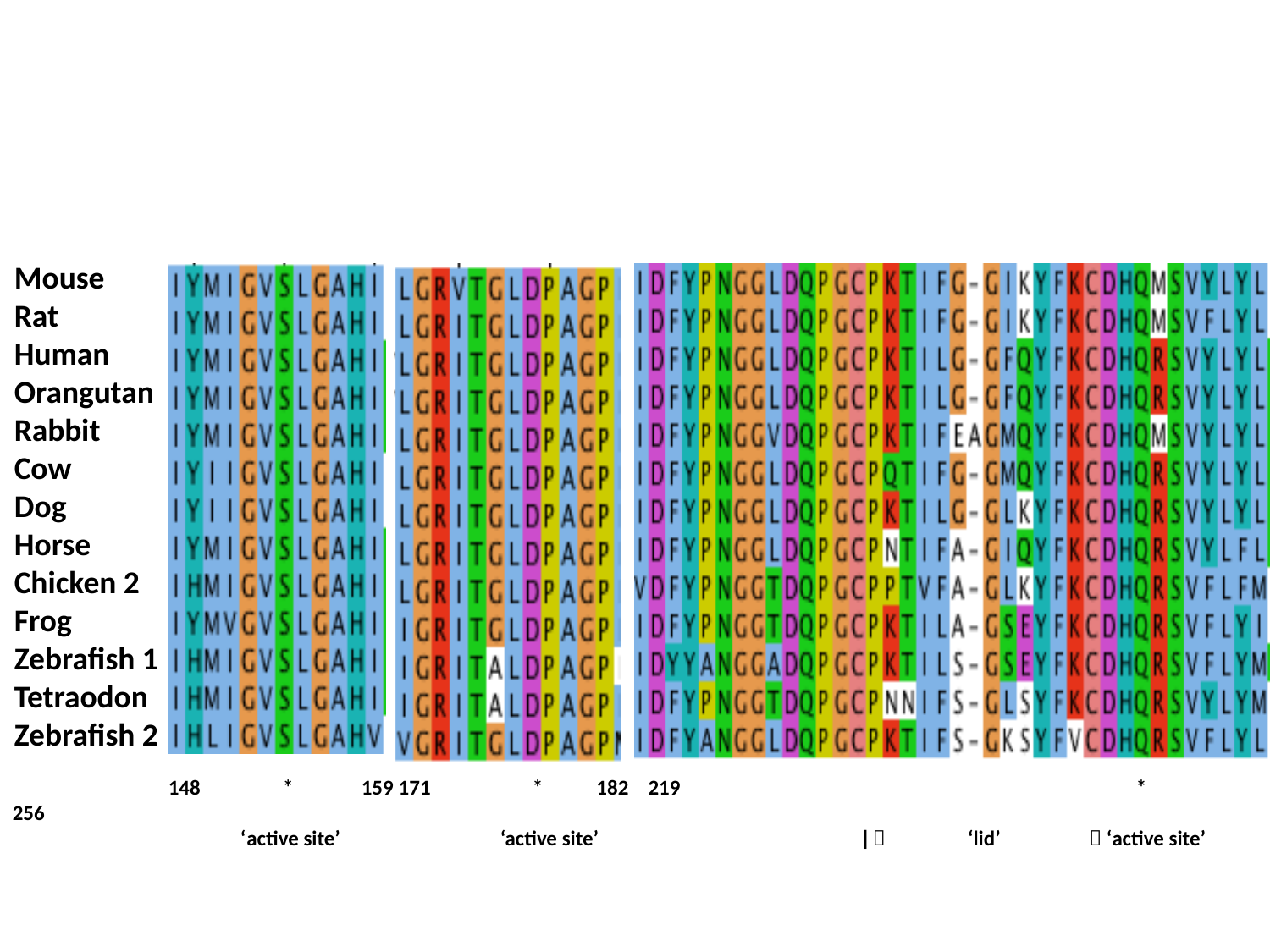

Mouse
Rat
Human
Orangutan
Rabbit
Cow
Dog
Horse
Chicken 2
Frog
Zebrafish 1
Tetraodon
Zebrafish 2
 148 * 159 171 * 182 219 * 256
 ‘active site’ ‘active site’ | ‘lid’  ‘active site’
