# Supplementary material for: Comparative structures and evolution of vertebrate lipase H (LIPH) genes and proteins: a relative of the phospholipase A1 gene families
Source: 3 Biotech. 2012 Sep 25;2(4):263–75. doi: 10.1007/s13205-012-0087-z (PMC3482443; doi:10.1007/s13205-012-0087-z)
Supplement: Supplementary file 2 — Supplementary material 2 (PPTX 110 kb) [file 13205_2012_87_MOESM2_ESM.pptx]

## Slide 1
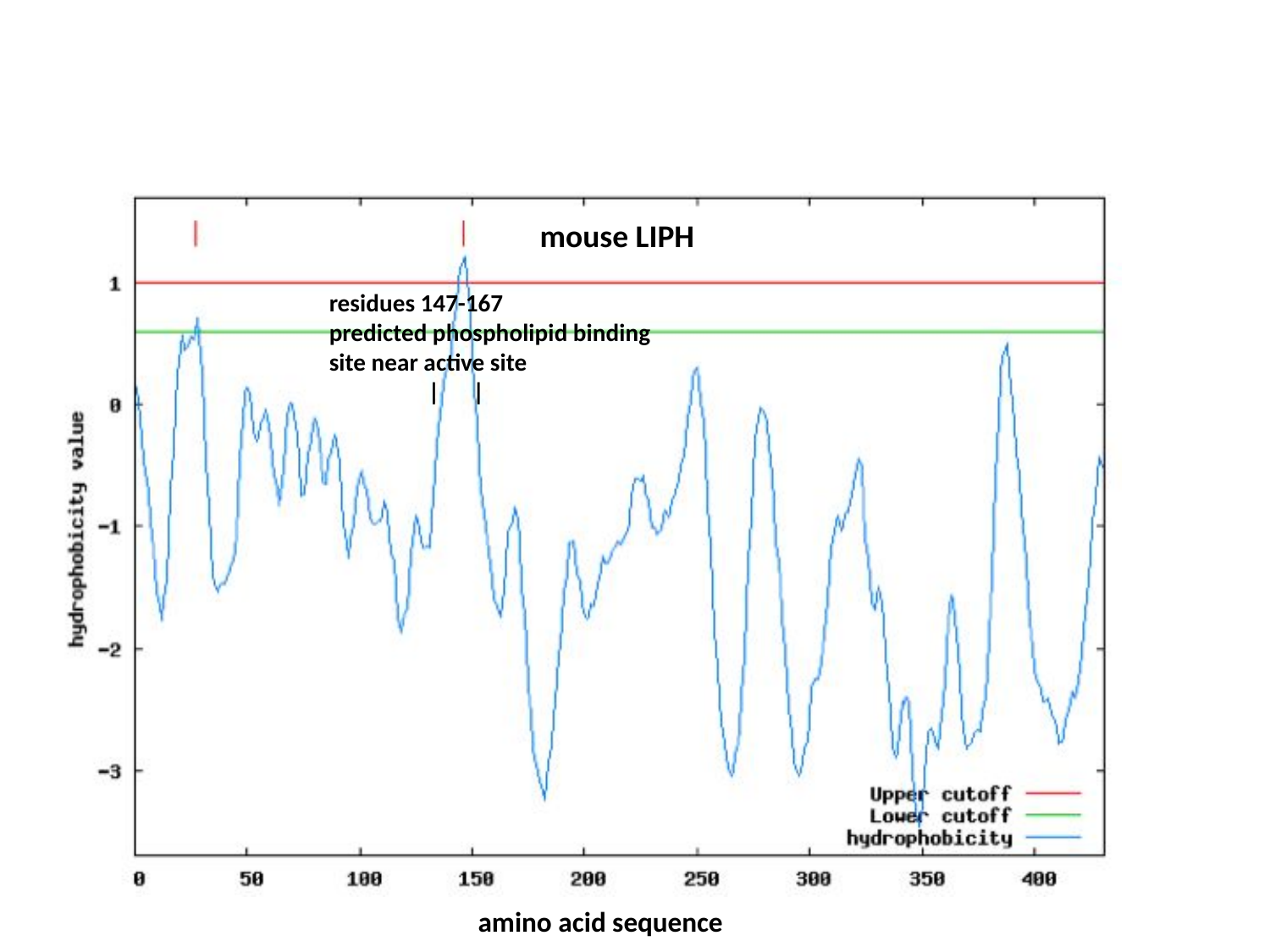

mouse LIPH
residues 147-167
predicted phospholipid binding
site near active site
 | |
amino acid sequence
