# Supplementary figures and images for: Comparative structures and evolution of vertebrate lipase H (LIPH) genes and proteins: a relative of the phospholipase A1 gene families
Source: 3 Biotech. 2012 Sep 25;2(4):263–75. doi: 10.1007/s13205-012-0087-z (PMC3482443; doi:10.1007/s13205-012-0087-z)

## Slide 1
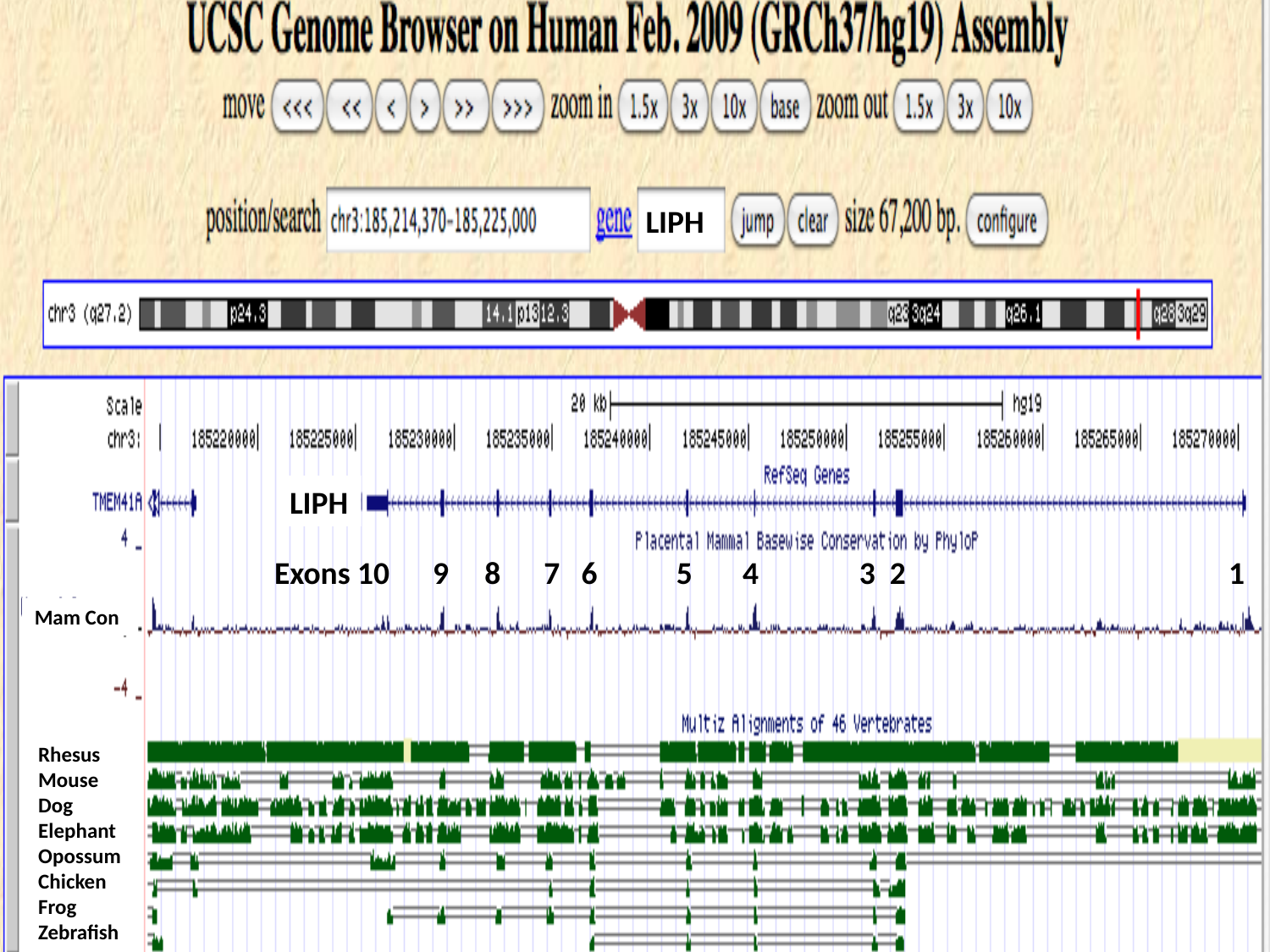

LIPH
LIPH
Exons 10 9 8 7 6 5 4 3 2 1
Mam Con
Rhesus
Mouse
Dog
Elephant
Opossum
Chicken
Frog
Zebrafish

Supplement: Supplementary file 3 — Supplementary material 3 (PPTX 238 kb) [file 13205_2012_87_MOESM3_ESM.pptx]

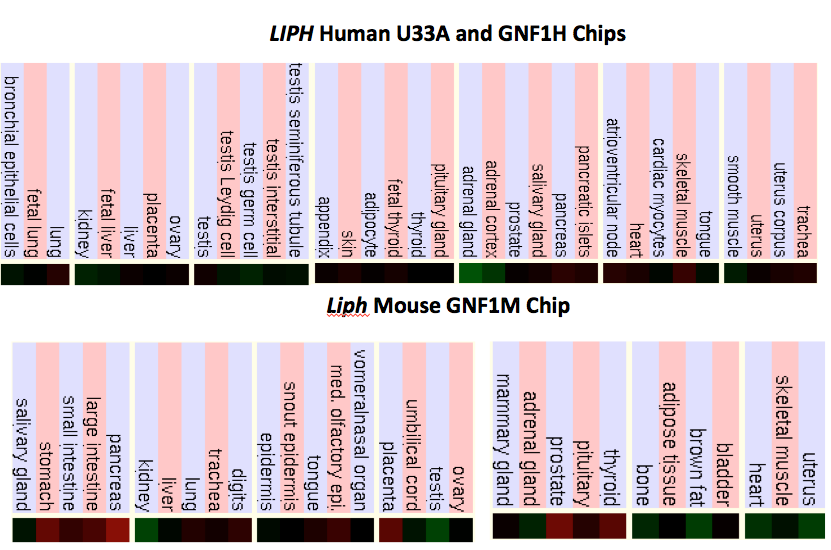

Supplement: Supplementary file 4 — Supplementary material 4 (PNG 124 kb) [file 13205_2012_87_MOESM4_ESM.png]
